# Properties of Loose-Fill Insulation Made of Leaves

**DOI:** 10.3390/ma19020425

**Published:** 2026-01-21

**Authors:** Christina Zwanger, Marcus Müller

**Affiliations:** Department of Material Development and Processing, University of Applied Forest Sciences Rottenburg, 72108 Rottenburg am Neckar, Germany; christina.sophia.zwanger@web.de

**Keywords:** bio-based insulation material, blow-in insulation, chopped leaf material, flame retardants, moisture properties, thermal properties, fire resistance

## Abstract

Urban leaf litter represents an underutilized biomass resource with potential applications in sustainable building materials. This study investigates the suitability of dried, comminuted leaves collected from municipal green areas as a loose-fill thermal insulation material. The material was characterized in terms of thermal conductivity, settlement behavior, fire reaction, resistance to mold growth, water vapor diffusion, hygroscopic sorption, and short-term water absorption. Tests were conducted following relevant DIN and ISO standards, with both untreated and flame-retardant-treated samples examined. Results indicate that the thermal conductivity of leaf-based insulation (λ = 0.041–0.046 W/m·K) is comparable to other bio-based loose-fill materials such as cellulose and wood fiber. Optimal performance was achieved for particles sized 2–16 mm, showing settlement below 1%. All variants, including untreated material, fulfilled the fire resistance requirements of class E, while selected treatments further improved fire resistance. The material exhibited moderate vapor permeability (μ ≈ 4–5), low water absorption, and moisture buffering behavior similar to that of other bio-based insulation materials. Resistance to mold growth was satisfactory under standardized conditions. Overall, the results demonstrate that leaf litter can serve as an effective and environmentally favorable loose-fill insulation material, offering an innovative recycling pathway for urban green waste.

## 1. Introduction

In 2022, the building sector and construction industry accounted for approximately 34% of global energy demand and 37% of CO_2_ emissions [1]. As such, this sector offers a high potential to contribute to the achievement of climate targets. Buildings serve to protect their occupants from environmental conditions such as heat, cold, wind, and moisture. Within this context, the choice of insulation materials plays a critical role in reducing energy losses during colder seasons and minimizing heat gain during warmer periods. Appropriate material selection during the construction phase can substantially reduce energy consumption over the building’s service life.

However, the environmental impacts of buildings are not limited to their operational phase. The production of construction and insulation materials is associated with considerable greenhouse gas emissions, and the end-of-life treatment of these materials also contributes to their overall footprint. Therefore, a comprehensive life cycle perspective is required—one that considers not only energy consumption and emissions during material production and building operation, but also the treatment, recycling or disposal of materials at the end of their life cycle.

Conventional insulation materials, such as expanded polystyrene (EPS), extruded polystyrene (XPS), mineral wool, and glass wool, are still largely disposed of through landfilling and thermally recovered, with only a small proportion being recycled currently [2]. In Germany, the insulation materials market is dominated by products derived from non-renewable resources. Although the share of insulation materials based on renewable resources has increased steadily over recent decades, it still represents only a minor proportion of the overall market [3].

Beyond commercially available renewable-based products, a wide range of alternative materials has been investigated for their insulation potential. In recent years, special attention has been given to agricultural residues and waste products. Bio-based materials, such as palm leaves, banana fibers, kenaf, kapok fibers, coconut fiber, sugarcane bagasse, pineapple leaves and sisal fiber, have been examined for their suitability as insulation material [4,5,6,7,8,9,10].

The primary objective in this context was to examine the suitability of these materials in insulation boards. Currently the most commonly used loose-fill insulation materials are wood fiber and cellulose. Other loose-fill materials include straw, wood shavings, seagrass, flax, leather, glass- and mineral wool [11]. Research over recent decades has explored a variety of additional loose-fill options, including wheat straw, corn stalk, and water reed, as well as agricultural byproducts like hemp shives, sunflower pith/bark, miscanthus stems, vine shoots, wheat chaff, maize husk, and mussel shells [12,13,14,15,16].

Leaves, which accumulate as a seasonal waste during autumn and early winter, represent an underexplored resource. Municipalities collect leaves from public green spaces and areas primarily for maintenance and traffic safety purposes. At present, most of this biomass is composted, with smaller proportions being incinerated or sent to landfill. A survey conducted as part of this study revealed a high potential for the utilization of this resource in Germany. The annual quantity of leaves collected by municipalities ranges from 620,000 to 740,000 tons [17,18].

Previous research has demonstrated the potential of leaf-based materials for use as thermal insulation. Vaivare et al. [19] investigated the thermal performance of apple tree leaves at varying densities and particle sizes, showing that both parameters significantly influence thermal conductivity. Similarly, Muizniece et al. [20] reported that pine and spruce needles exhibit favorable thermal insulation properties. In another study, Visockis [21] examined the thermal conductivity of a leaf–lime composite, further confirming the potential of plant-derived materials as sustainable insulation alternatives.

The use of leaf litter as a loose-fill insulation material has not been extensively explored or analyzed in depth. This study aims to fill that gap by evaluating the potential of leaf litter collected from urban green areas as an innovative, sustainable, and environmentally friendly alternative for thermal insulation. Leaf litter from urban green spaces is primarily processed in composting facilities and is otherwise not used for higher-value applications. Not only does this research contribute to such higher-value applications for an underutilized material, but it also aligns with growing efforts to reduce the environmental impact of traditional insulation materials.

Previous investigations into leaf material have largely focused on its thermal performance, specifically its thermal conductivity. However, these studies have overlooked other critical properties that influence the long-term viability and functionality of insulation materials. This research goes beyond thermal behavior by systematically assessing a wider range of key performance factors, including settlement and moisture behavior, mold growth resistance, and fire reaction. By expanding the scope of evaluation, this study offers a more comprehensive understanding of the suitability of leaf litter as a loose-fill thermal insulation material for practical applications.

## 2. Materials and Methods

Leaf material was provided by Technische Betriebe Rottenburg (TB Rottenburg, Rottenburg am Neckar, Germany) and collected during the autumn season 2022/2023 from green areas within the municipality of Rottenburg (Germany, Baden-Württemberg). Upon delivery, the material was immediately dried in wooden containers for two weeks using an air stream at a temperature of 25–30 °C. Following the drying process, the leaves were comminuted using a cutting mill. The resulting material was then fractionated with a tumbler (AS 400, Retsch GmbH, Haan, Germany) to obtain distinct particle size ranges. For production of bigger amounts of fraction 2–16 mm, a drum sieve (self-construction) was used.

### 2.1. Thermal Conductivity

The thermal conductivity of different particle sizes (0–0.5, 0.5–1, 1–2, 2–4, 4–6, 6–8, 8–10, 10–12, 0–16, 1–16, 2–16, 0–2, 2–6, 8–16, 16–31.5 mm and uncrushed) was measured to determine the influence of particle size on thermal insulation performance. The selection of these particle size fractions was guided by several considerations. Because thermal conductivity measurements are time-consuming, multiple experimental investigations were conducted in parallel. Consequently, the fractions chosen for thermal conductivity testing were adjusted based on findings from complementary experiments. For instance, preliminary processing trials revealed that material with particle sizes below 2 mm was difficult to handle due to excessive dust generation and was therefore excluded from further thermal conductivity measurements. The measurements were conducted according to DIN EN 12667 [22] at a mean temperature of 10 °C, using a guarded hot plate apparatus (GHP 500, NETZSCH-Gerätebau GmbH, Selb/Bavaria, Germany). Each particle size fraction was tested in duplicate, and average thermal conductivity values were recorded. Due to the wide range of particle sizes and their loose bulk density it was not possible to measure thermal conductivity at one specific bulk density. The specimens were prepared either by manually pouring the material loosely into the frame or by pneumatically filling polystyrene testing frames using a blowing machine (X-Floc-DS). The measuring field had a size of 250 × 250 mm^2^ and the thickness of the frame was 80 mm. Prior to filling, the material was conditioned at 23 °C and 50% relative humidity until a constant mass was reached to ensure moisture equilibrium.

### 2.2. Settlement Behavior

**Figure 1:**
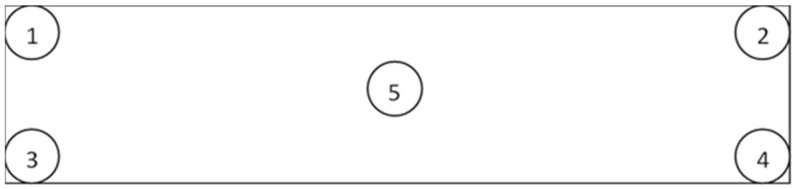
The circels with the numbers show the position of the measuring points on the top of the test box.

The settlement behavior was assessed according to DIN EN 15101-1 [23], method B.2. A particle size fraction of 2–16 mm was used for the test. The material was pneumatically filled into vertical test boxes with internal dimensions of 2300 mm (height), 600 mm (width) and 100 mm (thickness). Different machine parameters (air flow rate, number of blowers, material flow rate, airlock feed gate, type of filling tool: nozzle (X-floc Einblasdüse X-Jet 63, X-Floc Dämmtechnik Maschinen GmbH, Renningen, Germany) or pipe) were used to determine which delivers the least bulk density. For each parameter combination, 2 or 3 samples were tested. Due to limited resources, the material was not pre-conditioned prior to testing. The bulk density was calculated by weighing the filled specimen. Subsequently, the specimen was mounted vertically onto the test stand (Vibration Test Bench, X-Floc Dämmtechnik Maschinen GmbH, Renningen, Germany) and mechanical oscillation with a frequency of 50 s^−1^ was applied for a duration of 30 min. Settlement was calculated using the following equation:

(1) $$s=\frac{s_{i}-s_{1}}{s_{i}}\;\times 100,\;in\;\%$$

where S_i_ is the height of the insulation before applying the oscillation and S_1_ is the height of the insulation after applying the oscillation. The height was measured at five points on the top of the test box (Figure 1).

### 2.3. Reaction to Fire

Loose-fill insulation made of leaves treated with different fire retardants was tested according to DIN EN 11925-2 [24]. Leaf material of the fraction 2–16 mm was treated with ammonium phosphate (Kappaflam T4/729, Kapp-Chemie GmbH and Co. KG, Miehlen, Germany), boric salt (Coflam AB, Cofermin Chemicals GmbH and Co. KG, Essen, Germany), calcium hydroxide (Ca(OH)_2_, Carl Roth GmbH & Co. KG, Karlsruhe, Germany) and a mixture of sodium carbonate (Na_2_CO_3_, Carl Roth) and whey (sweet whey powder, myNatura, Bad Oeynhausen, Germany). Each treatment was applied at three concentrations (Table 1). All concentrations are given as percentage of the dry mass content of the leaves. All mixing procedures were carried out in a ploughshare mixer (Pflugscharmischer L20, Gebrüder Lödige Maschinenbau GmbH, Paderborn, Germany). Ammonium phosphate and the sodium carbonate–whey mixture were dissolved in deionized water and applied to the leaves by spraying. Boric salt and calcium hydroxide were incorporated by dry mixing with the leaves. After solution-based treatments, the leaves were dried at 70 °C for 50 to 100 h in a drying oven. The specimens were prepared by filling cages made of stainless-steel wire mesh, followed by conditioning at 23 °C, 50% relative humidity, until constant mass was achieved. For each treatment variant, five replicates were tested. Bulk density of the specimens ranged between 95 and 130 kg/m^3^. During testing, specimens were exposed to a flame for 30 s, followed by an observation period of 30 s.

### 2.4. Resistance to Mold Growth

The resistance to mold growth was evaluated using two standardized test methods: DIN EN 17886:2024-03 [25] and EAD 040138-01-1201 [26].

#### 2.4.1. DIN EN 17886:2024-03

Material from all variants as defined in the reaction to fire test was sterilized using gamma irradiation. For each formulation, three replicates were prepared. Therefore, the sterilized material was placed in petri dishes. The inoculum contained four of the six fungal strains required by the standard: *Aspergillus versicolor* DSM 63292, *Chaetomium globosum* DSM 1962, *Talaromyces pinophilus* DSM 1944, and *Trichoderma viride* DSM 63065. All strains were obtained from the Leibniz Institute DSMZ (German Collection of Microorganisms and Cell Cultures GmbH, Braunschweig, Germany). *Aspergillus niger* (DSM 1957) and *Paecilomyces variotii* (DSM 1961) were excluded due to their classification in risk group 2 (TRBA 460). As the laboratory facilities at the University of applied Forest Sciences in Rottenburg are certified only for Biosafety Level 1 (S1), these organisms could not be used. The inoculum was applied by spraying the suspension on the prepared petri dishes. Following inoculation, specimens were incubated in a climate chamber at 28 °C and 80% relative humidity until the validity criteria of the standard were met. The results were evaluated according to Table 2 of DIN EN 17886:2024-03.

#### 2.4.2. EAD 040138-01-1201

Sample material was filled in cages made of stainless steel with dimensions of 50 × 50 × 30 mm^3^ (length × width × height). The bulk density of each material was adjusted according to the intended application density. The samples were prepared and placed in a desiccator which was filled with deionized water at the bottom (approx. 20 mm). For this test, a reduced set of variants was examined (A15, B15, K15, MS6/3). In addition, a second variant of the untreated leaves was included. This reference material (RW) was washed twice with deionized water before testing, with the aim of reducing the load of naturally occurring spores. The desiccators were stored in a climate chamber at 23 °C for four weeks. The results were evaluated in accordance with Table 4 of EN ISO 846:1997 [27].

### 2.5. Water Vapor Diffusion Resistance Factor

The water vapor resistance factor *µ* was determined according to DIN EN 12086 [28]. The specimens were prepared by filling the material into circular cups with a diameter of 237.6 mm and a height of 110 mm using a blowing machine. The cups were closed with a stainless-steel mesh at the bottom (mesh size 1 mm). The following material variants were examined: R, A15, B15, K15, MS6/3. For each variant five samples were prepared. Measurement was carried out in accordance with Set A (DIN EN 12086, Table 1) using the “dry cup” method. The specimens were conditioned in a climate chamber at 23 ± 0.5 °C, with a relative humidity of 50%. The mass change was monitored daily by weighing the specimens in intervals of 24 h. Bulk density was determined for each specimen. The water vapor permeability *δ_a_* was calculated using the following equation:

(2) $$\delta_{a}=\;\frac{0.086\times p_{0}}{R_{D}\times T\times p}\;\times {\left(\frac{T}{273}\right)}^{1.81}$$

where R_D_ is the gas constant of water vapor 462.10^−6^ Nm/(mg*K), T is the thermodynamic temperature in K, p_0_ is the atmospheric pressure (1013.25 hPa) and p is the mean atmospheric pressure in the test chamber.

### 2.6. Short Term Water Absorption by Partial Immersion

The measurements were performed according to DIN EN ISO 29767 [29]. The material variants listed in Table 1 were examined. For each variant, four specimens were prepared by blowing the material in stainless steel cages (200 × 200 × 155 mm^3^, length × width × height) made of wire mesh with a mesh size of 10 mm. To prevent material loss during testing, the cages were additionally lined with an inlay made of fine mesh (1 mm). The examination was carried out using method A. The short-term water absorption, W_p_ (kg/m^2^), was then calculated from the following equation:

(3) $$W_{p}\;=\;\frac{m_{24}-\;m_{0}}{A_{p}}$$

where m_0_ is the initial mass, m_24_ the mass after 24 h (±30 min) of partial immersion and A_p_ is the bottom surface area.

### 2.7. Hygroscopic Sorption Properties

The hygroscopic sorption properties were determined according to DIN EN ISO 12571:2020-11 [30]. Five specimens of each variant with a mass between 13.1 and 14.9 g were measured. In this test, the climate chamber method was chosen. The specimens were placed in the climate chamber at a temperature of 23 ± 0.5 °C and relative humidity of 30, 40, 50, 60, 70, 80%. Moisture content was calculated according to the following equation:

(4) $$u=\;\frac{m-m_{0}}{m_{0}}$$

where m_0_ is the oven-dry mass and m is mass at the respective relative humidity.

### 2.8. Statistical Analysis

Statistical analyses were conducted using R software (RStudio 4.5.1, Posit PBC). The properties evaluated included reaction to fire, water vapor resistance factor, and short-term water absorption. For all measured parameters, the Shapiro–Wilk test was used to assess data normality. When normal distribution could not be confirmed, non-parametric comparisons were performed using the Wilcoxon rank-sum test, with statistical significance set at *p* < 0.05.

## 3. Results

### 3.1. Thermal Conductivity

**Figure 2:**
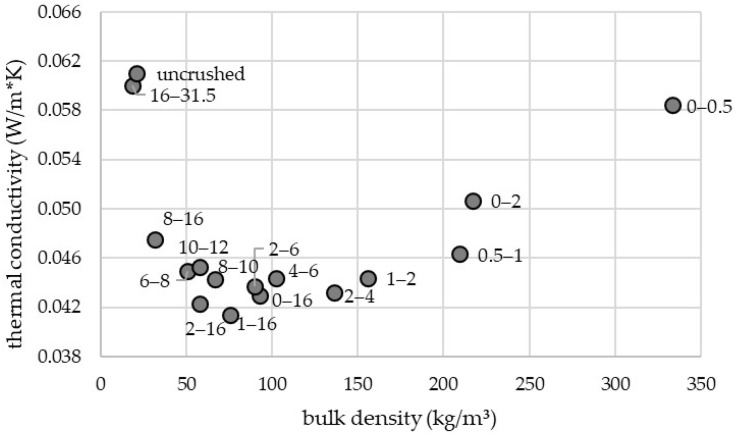
Thermal conductivity and associated density of different particle sizes and particle size ranges of test specimens produced by loose filling (Table 2).

To identify the particle size distribution associated with minimal thermal conductivity, multiple particle size fractions were evaluated using a guarded hot plate apparatus. Figure 2 depicts the measured thermal conductivity as a function of bulk density for the investigated fractions. Test specimens were prepared by loosely filling the material into the testing frame. The particle size ranges 0–16 mm, 1–16 mm, 2–16 mm, 2–4 mm, 2–6 mm with bulk densities of 93, 76, 58, 136, 90 kg/m^3^, respectively, showed the lowest thermal conductivity values (Table 2).

**Figure 3:**
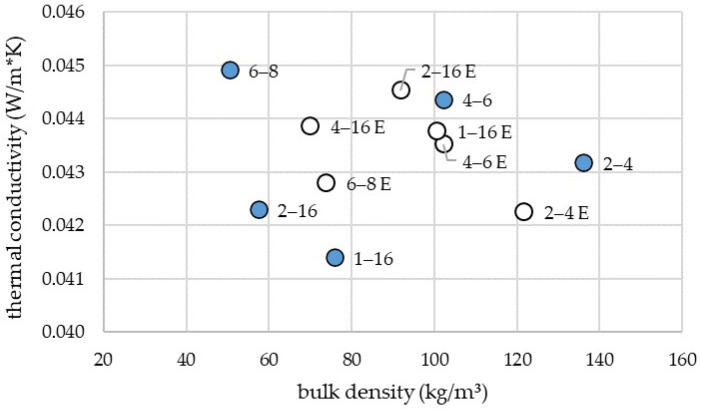
Comparison of the thermal conductivity and density of different particle sizes of test specimens produced either by loose filling (blue) or with the aid of a blowing machine (white).

Figure 3 presents the thermal conductivity and corresponding bulk density for the 2–4 mm, 4–6 mm, 6–8 mm, 1–16 mm and 2–16 mm fractions. Table 3 presents the values of thermal conductivity shown in Figure 3. Specimens were prepared using two different methods: loose filling (blue markers) and pneumatic blowing using an insulation blowing machine (white markers). With the exception of the 2–4 and 4–6 mm fraction, all size ranges exhibited higher bulk densities when prepared by pneumatic blowing. However, an increased density did not invariably result in increased thermal conductivity, as evidenced by the 4–6 mm and 6–8 mm fractions. For the 2–4 mm, 1–16 mm, and 2–16 mm fractions, a positive correlation between bulk density and thermal conductivity was observed. For these fractions, a higher density resulted in higher thermal conductivity.

Based on the results of Figure 2 and the experience gained during processing of the material, the particle size range of 2–16 mm was selected for subsequent testing. Although the 0–16 mm and 1–16 mm fractions displayed slightly lower thermal conductivity, their processing generated considerable dust, resulting in elevated occupational exposure. The 2–16 mm fraction was therefore selected as the most suitable compromise between thermal performance and safe handling. In addition to evaluating different particle size fractions and application methods, leaf material within the 2–16 mm fraction treated with various flame retardants (Table 1) was also examined. The results showed that the flame-retardant treatments had no measurable effect on thermal conductivity.

### 3.2. Settlement Behavior

**Figure 4:**
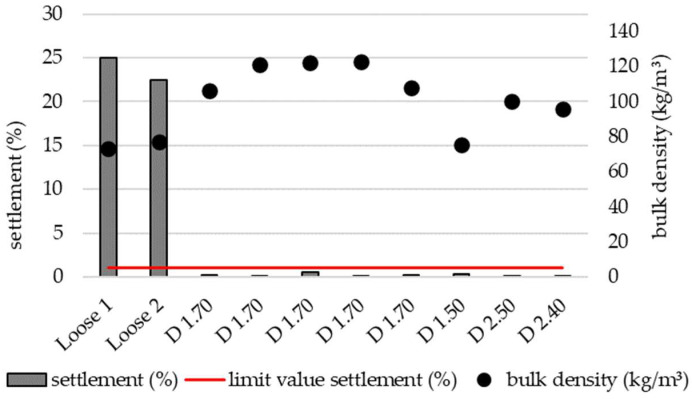
Settlement and corresponding bulk density of specimens prepared by two methods: loose filling without an insulation blowing machine (Loose 1 and Loose 2) and pneumatic blowing using an insulation blowing machine with a nozzle (D1.70–D2.40).

**Figure 5:**
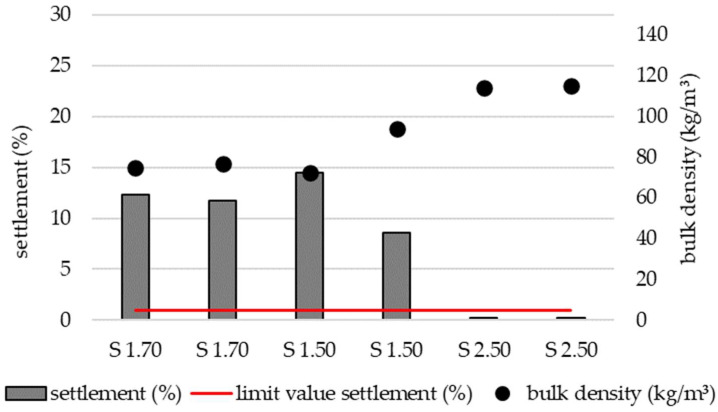
Settlement and corresponding bulk density of specimens prepared by pneumatic blowing using an insulation blowing machine with a pipe as filling tool.

The settling behavior was examined by testing various setting parameters on the blowing machine. The aim was to achieve a settling rate of less than 1%, as this is the minimum requirement specified in DIN 4108-10 [31]. The adjustable parameters included the type of filling tool (nozzle or pipe), number of blowers, air flow rate, material flow rate, and feed gate opening. The designation of the test specimens is structured as follows: the letter indicates whether the specimen was filled using the nozzle (D) or the pipe (S), the first digit specifies the number of blowers used (one or two), and the second digit indicates the air flow rate as a percentage. Figure 4 and Figure 5 present the results of these trials.

Preliminary tests revealed that a minimum air flow rate of 40% was required to transport material through the pipe; lower flow rates resulted in clogging of the pipe or nozzle. The material flow rate was maintained at 50% for all tests, as this setting—combined with air flow rates between 40% and 80% (with 1 or 2 blowers)—yielded the most stable processing conditions. The feed gate opening was fixed at setting 5 in all experiments.

Loose 1 and Loose 2 represent specimens in which material was manually filled into the test frame, thereby simulating a worst-case scenario. These specimens exhibited the highest settlement values at 22.5% and 25.0%, respectively. By adjusting the air flow rate and number of blowers, bulk density—and consequently settlement behavior—could be effectively controlled. Settlement below 1% was achieved with air flow rates between 40% and 80%, using either one or two blowers and both filling tools (nozzle and pipe). The lowest bulk density associated with a settlement below 1% was 75 kg/m^3^, achieved with one blower, an air flow rate of 50%, and the nozzle filling method. However, under these conditions, nozzle clogging occurred frequently, indicating limited practical applicability for real-world installation.

A comparison of the results presented in Figure 4 (nozzle) and Figure 5 (pipe) demonstrates that identical machine settings produced different settlement behaviors depending on the filling method. For example, when comparing D1.70 with S1.70 and D1.50 with S1.50, only specimens prepared using the nozzle achieved settlement below 1%. Samples prepared with one blower, an air flow rate of 70%, and the nozzle exhibited higher bulk density compared to samples prepared with the pipe under identical settings. Conversely, at an air flow rate of 50%, specimens prepared with the nozzle displayed slightly lower bulk density than those prepared with the pipe.

### 3.3. Reaction to Fire

**Figure 6:**
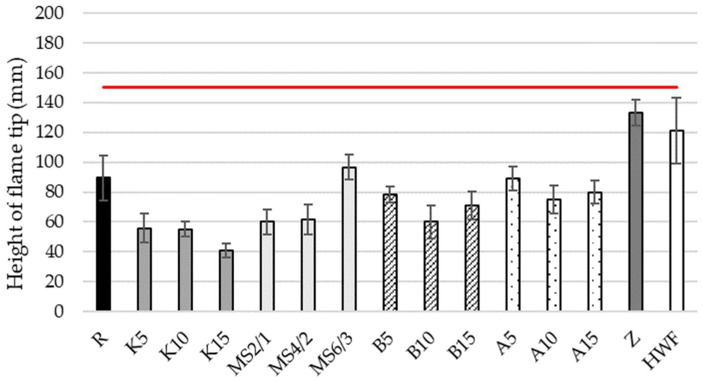
Flame-damaged height after 30 s during single-flame source test, compared with commercial cellulose (Z) and wood fiber (HWF) insulation. The red line indicates the requirement height of class E.

To evaluate whether the leaf-based loose-fill insulation complies with fire resistance class E, a single-flame source test was performed in accordance with the relevant standard. Figure 6 presents the results for untreated material (R), material treated with various flame retardants at different concentrations (K5–A15), and, for comparison, commercial cellulose insulation (Z) and wood fiber insulation (HWF).

As shown in Figure 6, all developed loose-fill samples met the requirements for fire resistance class E. Compliance with class E would already have been achieved with a flame exposure duration of 15 s. Nevertheless, to assess the potential for achieving a higher classification (class D), the specimens were exposed to direct flame for 30 s.

The variants K5, K10, K15, MS2/1, MS4/2, B10, and B15 exhibited a significantly lower flame tip height compared to the untreated reference material (R). In contrast, MS6/3, B5, A5, A10, and A15 showed no statistically significant difference from the untreated material. When compared with the cellulose insulation samples, all leaf-based materials—both treated and untreated—demonstrated significantly lower flame tip heights. Relative to the wood fiber insulation, all leaf-based materials except MS6/3 also exhibited significantly lower flame tip heights.

### 3.4. Resistance to Mold Growth

Thirteen variants of leaf material with a particle size of 2–16 mm, both untreated and treated with various flame retardants at different concentrations, were evaluated in accordance with DIN EN 17886 [25]. In addition to visual inspection, quantitative assessment was performed by counting cultivable fungal units (CFUs). The results are summarized in Table 4.

Untreated material (R), as well as material treated with ammonium phosphate, boric salt, and the whey–sodium potassium mixture at the lowest concentration, were classified as class 0 (no visible mold, either to the naked eye or under microscopic observation). Despite this classification, mold development was observed, as indicated by an initial mold count of approximately 4.0–4.3 log_10_ CFU/cm^3^ at the beginning of the test. By the end of the test, the number of cultivable molds was slightly higher (by 0.1 log_10_ CFU/cm^3^), unchanged, or slightly lower (by 0.1–0.2 log_10_ CFU/cm^3^), depending on the variant.

Material treated with calcium hydroxide and with the whey–sodium potassium mixture at the second- and third-highest concentrations was classified as class 1a (no visible mold to the naked eye, but microscopic evidence of mold covering up to 25% of the test surface). These samples also exhibited initial mold counts of approximately 4.0–4.2 log_10_ CFU/cm^3^. At the end of the test, CFU counts were again either slightly higher (by 0.1 log_10_ CFU/cm^3^), unchanged, or slightly lower (by 0.1 log_10_ CFU/cm^3^).

For comparison, resistance to mold growth was additionally assessed following EAD 040138-01-1201 (Table 5). The tested variants included untreated material (R), untreated material washed twice (RG), material treated with 5% boric salt (B5), seaweed-based material (SG), straw insulation (S), cellulose insulation (Z), and wood fiber insulation (HWF). Under these test conditions, R, RG, B5, SG, and S were classified as class 2 (mold visible to the naked eye, up to 25% surface coverage), whereas Z and HWF were classified as class 0 (no mold visible to the naked eye or under microscopic observation).

### 3.5. Water Vapor Diffusion Resistance Factor

The water vapor diffusion resistance factor was measured for leaf material with a particle size of 2–16 mm, both untreated and treated with various flame retardants at different concentrations. As Table 6 shows, the highest water vapor diffusion resistance factor was observed for leaf material treated with ammonium phosphate. The lowest water vapor diffusion resistance factor was observed for leaf material treated with calcium hydroxide. Untreated material and material treated with the mixture of whey and sodium potassium and boric salt had vapor diffusion resistance factors between 4.4 and 5.2.

### 3.6. Short Term Water Absorption by Partial Immersion

The measurement results for untreated material (R) and material treated with various flame retardants at different concentrations are summarized in Table 7. The untreated reference exhibited the highest short term water absorption, with a mean value of 5.3 ± 0.95 kg/m^2^. The lowest short term water absorption with 2.2 kg/m^2^ was observed for leaf material treated with 10% calcium hydroxide (K10). Overall, all treated variants showed slightly lower short term water absorption compared to the untreated reference. Statistically significant reductions in short term water absorption were observed for the variants K5, K10, MS2/1, B5, B10, B15, and A5, whereas all remaining variants were not statistically different from the untreated material.

### 3.7. Hygroscopic Sorption Properties

Table 8 summarizes the results of water content measurements for untreated material and material treated with various flame retardants at different concentrations. For each material, the equilibrium water content (%M) was determined at relative humidities of 30%, 50%, 60%, 70%, and 80%. Across all relative humidity levels, only minor differences in water content were observed between the treated and untreated materials. An exception was noted for variant A15, which exhibited an unusually high water content of 18.8% at 80% relative humidity, compared with 15.1% for the untreated reference and 14.2–15.7% for all other treated variants.

## 4. Discussion

### 4.1. Thermal Conductivity

There are four modes of heat transfer in insulation materials: conduction through the solid medium, conduction through the gas medium, i.e., the air trapped between the particles, convection due to the air in the space between the particles, and radiation interchange between particles and air [32,33,34]. The relative contribution of these mechanisms depends strongly on the pore size distribution, particle geometry, and bulk density.

Figure 2 demonstrates that the thermal conductivity of the leaf material is influenced by both particle size and bulk density. The results indicate a non-linear relationship: thermal conductivity increases at both very low and very high densities, while an optimum range exists at intermediate densities where the balance between heat transfer through the solid phase, the gas phase, and convection minimizes overall thermal conductivity. Larger particle sizes (e.g., uncrushed material, 16–31.5 mm or 8–16 mm fractions) exhibited higher thermal conductivity due to the larger pore volumes, which enhance heat transfer through the gas phase and promote convective processes. Conversely, very fine particle sizes (e.g., 0–0.5 mm, 0–2 mm, or 0.5–1 mm fractions) also showed increased thermal conductivity, as the reduced pore volume results in a greater number of particle–particle contact points, thereby intensifying heat transfer through the solid phase. Comparable trends have been reported in previous studies. Schunk et al. [35] observed increasing thermal conductivity with decreasing bulk density in milling chips derived from different wood species. Similar effects were described by Martínez-García et al. [12] and Kosiński et al. [36]. Ruckdeschel et al. [37] examined the thermal conductivity of silica hollow nanosphere assemblies and demonstrated that smaller particle sizes reduce pore space, thereby diminishing the Knudsen effect, while stronger interparticle bonding enhances solid conduction, collectively resulting in increased thermal conductivity.

Figure 3 presents the results for samples of the same particle size, which were either prepared by loosely filling in or prepared with the aid of a blowing machine. For fractions of 1–16, 2–15 and 6–8 mm, preparing the samples with a blowing machine led to a higher density. This could be due to the pressure which is applied through pneumatically transporting the leaf particles into the testing frame. For fraction 4–6, no difference in density could be observed. Fraction 2–4 showed a lower density when applied by loosely filling in. This suggests that filling with the blowing machine causes the particles to be arranged in a different structure than when filling loosely.

### 4.2. Settlement Behavior

Dynamic loads occurring during the transport of prefabricated wall components and during the service life of buildings can generate vibrations that cause loose-fill insulation materials to settle. Such settlement may adversely affect the thermal performance of building envelopes, as the formation of cavities can create thermal bridges, while compaction of the insulation material alters its thermal behavior. According to Heisel et al. [38], the settlement behavior of loose-fill insulation materials is strongly dependent on installation density; higher densities generally result in reduced settlement. Several parameters have been identified influencing the settlement of loose-fill insulation, including the type of bulk material, the geometry of the cavity to be filled, the applied force and vibration frequency, climatic conditions, and the installation method [39,40]. To minimize settlement, several strategies can be employed, such as installing the material at sufficiently high density, dividing the cavity height with intermediate supports (“bridges”), or developing lightweight and resilient particle structures. Teslík et al. [41] achieved zero settlement for blown-in straw insulation at a bulk density of 120 kg/m^3^. In the present study, settlement below 1% was achieved at densities between 75 kg/m^3^ and 120 kg/m^3^. Notably, measurement D1.50 exhibited an exceptionally low bulk density of 75 kg/m^3^, which may be attributed to variations in particle size distribution. Further measurements are recommended to confirm this finding. Alternatively, the remarkably low settlement could result from the particular arrangement of particles formed during the blowing-in process, which differs from the structure observed in manually filled samples (Loose 1 + 2) of comparable density that exhibited settlements of 22–25%. Comparable settlement behavior of loosely filled insulation were reported by Svennerstedt B. [42], who studied the long-term settlement of cellulose and mineral fiber insulation in timber frame walls. After 36 months, he observed settlements of 18% for cellulose and 4.5% for mineral wool. Rojas-Herrera et al. [43] investigated the use of sawdust waste from advanced manufacturing as loose-fill insulation applied with a blowing machine (Minifant M99-DS). Using an air flow rate of 40%, a material flow rate of 100%, a feed gate opening of 1, and a nozzle application, the lowest achieved density was 123.77 kg/m^3^. However, the study did not report settlement behavior, preventing a direct comparison with the results obtained for leaf-based insulation in the present study.

### 4.3. Reaction to Fire

According to several studies, calcium-based materials can be a promising alternative flame retardant, as they have a high suppression ability, are renewable and available at low cost. Lv et al. [44], Fluegeman et al. [45], Koshiba et al. [46], Hamdani-Devarennes et al. [47], and Laoutid et al. [48,49] examined different calcium-based flame retardants and showed their potential. The findings from these studies support the results shown in Figure 6, where the variants treated with calcium hydroxide exhibited a significantly lower flame tip height compared to the untreated reference material.

Whey protein has also been identified as an effective bio-based flame retardant. Previous studies have demonstrated that whey proteins can reduce the burning rate and prolong total burning time through a combination of mechanisms: formation of an oxygen barrier, inhibition of oxygen diffusion, high moisture content leading to heat absorption, and enhanced char formation [50,51,52]. The results of the present study confirm these observations for variants MS2/1 and MS4/2, both of which showed improved fire performance. However, variant MS6/3, containing the highest proportion of whey protein in the flame-retardant formulation, did not show a significant difference compared to the untreated reference, indicating that excessive protein content may negatively affect flame retardancy or interfere with the coating homogeneity.

Andzs et al. [14] used a mixture of 7% sodium tetraborate and 8% boric acid to treat wheat straw, water reed and corn stalk prepared through chopping, steam explosion, or chemo-mechanical pulping. In the single-flame source test following ISO 11925-2:2020 [24], all treated samples—except the steam-exploded water reed—achieved flame-damaged heights below 150 mm, fulfilling the requirements for fire resistance class E. However, the study did not include untreated control materials, making it impossible to quantify the effect of the treatment.

Inorganic salts such as ammonium phosphate are widely used as flame retardants for lignocellulosic materials, particularly wood fiber. Previous studies have demonstrated that the application of monoammonium phosphate (10 wt%) can markedly enhance the fire resistance of wood fiber insulation [53]. The ammonium phosphate employed in the present study was specifically formulated for use with wood fiber materials, according to the supplier’s specifications. However, as shown in Figure 6, the variants treated with ammonium phosphate (A5, A10, and A15) did not exhibit a statistically significant improvement in fire retardancy compared to the untreated reference. During sample preparation, segregation between the flame retardant and the leaf particles was observed. This may have led to a low content of fire retardant in the examined material and contributed to the absence of measurable differences in fire performance between treated and untreated samples.

Comparable to the untreated leaf material in the present study, crushed straw exhibits similar behavior under the single-flame source test. Teslík [54] and Teslík et al. [55] demonstrated that untreated crushed straw can also be classified within fire reaction category E, supporting the observed fire behavior of untreated leaf-based materials.

Conventional loose-fill insulation materials, such as cellulose and wood fiber, typically do not meet the requirements of fire resistance class E without the incorporation of flame retardant additives [53,56,57,58]. In contrast, the present study demonstrates that certain bio-based materials—such as the investigated leaf material and crushed straw—can achieve the required fire resistance class without the addition of flame retardants. This intrinsic fire resistance represents a considerable advantage for such materials, both from an environmental and an economic perspective.

### 4.4. Resistance to Mold Growth

A wide variety of test methods exists to assess the resistance of insulation materials to mold growth. Several authors have highlighted the lack of standardization and the resulting challenges in comparing results across studies [59,60]. Methodological differences include the type of fungal species used, the inoculation procedure, test climate, exposure duration, analytical techniques, and the criteria for visual or quantitative evaluation. Imken et al. [61] reported substantial variability in outcomes depending on the selected test method, emphasizing the need for methodological harmonization. Like many organic insulation materials, leaf-based insulation is hygroscopic and therefore sensitive to microbial colonization by fungi and bacteria. The microbial quality of the leaf material, similar to that of other industrial crops such as flax, hemp, and straw, is strongly influenced by atmospheric conditions during growth and the collecting of the material, which can result in a high diversity of naturally occurring mold species [61]. The critical moisture level for mold growth on building materials was examined by Johansson [62]. For mineral wool insulation and other insulation materials, this critical moisture level is 90–95% relative humidity.

In the present study, using DIN EN 17886, the specific fungal strains applied did not grow on either the untreated leaf material or on the material treated with boric salt or ammonium phosphate. Since no growth was observed on the reference material, it cannot be concluded that these additives exerted a fungicidal effect. Nevertheless, previous research has demonstrated that boric acid and borates act as effective biocides in cellulose-based insulation materials [63,64].

The samples treated with calcium hydroxide and with the whey–sodium–potassium mixture showed a tendency toward increased mold growth. Mitterböck and Korjenic [65] reported that slaked lime (Ca(OH)_2_) had a positive influence on straw and sheep wool insulation by acting as a moisture buffer, which helped prevent mold formation. In contrast, the present study did not observe a moisture-reducing effect in the leaf material treated with calcium hydroxide (Table 5), which may explain the different results.

Treml [66] investigated mixtures of untreated wood shavings and cellulose—both untreated and treated with 8% boric salt—to assess their resistance to mold growth using a method comparable to DIN EN 17886. The study demonstrated that mixtures containing untreated wood shavings and untreated cellulose supported fungal growth, whereas mixtures incorporating boric-salt-treated cellulose exhibited no fungal development and were classified as class 0. These findings are consistent with the results of the present study, in which leaf-based samples treated with boric salt also achieved class 0, indicating inhibition of fungal growth.

When tested according to EAD 040138-01-1201, the untreated leaf material (R and RG), the boric salt-treated variant (B5), chopped seaweed (SG), and loose-fill straw insulation (S) all exhibited mold growth classified as Class 2 according to DIN EN ISO 846:1997. Koh et al. [67] assessed the mold resistance of wheat and barley straw using 4% boric acid, following EAD 040005-00-1201 [68], Annex B, and found heavy mold growth in untreated straw. Although boric acid delayed fungal colonization, it did not fully prevent it—consistent with the findings of the present study, where no significant difference was observed between untreated (R) and boric salt-treated (B5) leaf material. Similarly, Soto et al. [69] reported fungal colonization of untreated wheat straw within 4–5 days at 30% relative humidity, accompanied by mass losses of 13% for straw and 3.8–4.3% for cellulose treated with boron salts. Comparable results were obtained by Marques et al. [70], who examined rice straw and classified untreated samples as class 3, with fungal growth visible to the naked eye covering up to 50% of the surface.

Comparing the results of DIN EN 17886 (Method 1) and EAD 040138-01-1201 (Method 2) reveals clear methodological effects. In Method 1, neither the untreated reference nor the boric salt-treated material showed fungal growth, whereas both materials developed mold in Method 2. This difference can be attributed to the sterilization and inoculation procedures: Method 1 used sterilized material inoculated with defined fungal strains, while Method 2 employed unsterilized samples, allowing naturally occurring microorganisms to remain active. These observations indicate that boric salt may exhibit selective antifungal efficacy, being effective against specific inoculated strains but not against the diverse natural microflora inherently present in leaf material. Consequently, assessing the biological durability of bio-based insulation materials requires careful consideration of test conditions, as sterilization and fungal selection can significantly influence the outcomes.

### 4.5. Water Vapor Resistance Factor

The water vapor diffusion resistance factor (μ-value) is a fundamental parameter describing the permeability of a building material to water vapor in relation to air. It characterizes the resistance a material offers to vapor diffusion and is therefore an essential indicator of its hygrothermal performance. A low μ-value indicates high vapor permeability, which is advantageous when humidity gradients between indoor and outdoor environments increase, as it helps to reduce the risk of condensation and subsequent mold growth [71]. Preventing moisture accumulation within building components is critical, since the thermal conductivity of insulation materials rises with increasing moisture content. Consequently, insulation layers should exhibit good vapor permeability, achieved through materials with low vapor diffusion resistance.

Berzins et al. [72] investigated the vapor diffusion properties of chopped and chemimechanically pulped wheat straw, water reeds, and corn stalks. The chopped fractions are structurally comparable to the leaf material analyzed in the present study. For these chopped plant materials, vapor diffusion coefficients ranging between 3.1 and 3.3 were reported, which are slightly lower than the value measured for untreated leaf material (μ = 4.7). This deviation may be attributed to the higher bulk density of the leaf specimens. A positive correlation between density and the water vapor resistance factor has also been described by Marques et al. [70] and Lebed and Augaitis [73]. Similarly, Fedorik et al. [62] observed an increase in μ-value with increasing density for loose-fill insulation materials composed of moss, recycled paper, cutter chips, feathers, and peat–sphagnum mixtures, although the peat-based insulation maintained high permeability even at elevated densities. Nevertheless, it was demonstrated that this correlation does not apply to all materials: the peat-based insulation allowed the highest permeability regardless of the high density.

Fibrous insulation materials such as glass wool, rock wool, expanded polystyrene, wood fiberboard, and polyester fiberfill typically exhibit low vapor diffusion coefficients (μ ≈ 2), a characteristic attributed to their open fibrous structure [74]. For hemp-based insulation, Collet et al. [75] measured μ-values of 3.6–3.7 for hemp wool with an organic binder (HW1) and for hemp wool reinforced with cotton fibers and a polyester binder (HW2), using the dry cup method. Jiřičková and Černý [76] reported μ-values of 4.2 at 5/26% relative humidity and 3.7–3.9 at 5/97% relative humidity for hydrophilic, hydrophobic, and unmodified mineral wools—notably higher than the typical μ-value of ≈1 provided in commercial data sheets for standard mineral wool products.

Koh et al. [67] examined the vapor diffusion resistance of wheat and barley straw treated with boric acid and found no substantial difference between treated and untreated materials. Comparable findings were obtained in the present study, where no significant differences in vapor resistance were observed between the untreated reference and the materials treated with whey–sodium–potassium mixture, calcium hydroxide, or boric salt. In contrast, the sample treated with ammonium phosphate exhibited a 2.8-fold higher water vapor resistance factor compared to the untreated material, indicating a potential densification or structural alteration effect caused by the treatment.

### 4.6. Short Term Water Absorption by Partial Immersion

According to DIN EN 1609:2013 [77], this experimental method simulates the water uptake of materials during a 24-h rain period that may occur in the construction phase. A low short term water absorption (W_p_) is desirable, as it indicates reduced moisture uptake and consequently a lower risk of moisture-induced damage during installation. Water absorption is primarily influenced by the hygroscopicity and porosity of the material. At higher bulk densities, the greater amount of solid material provides more capacity for water absorption. Conversely, materials with lower densities tend to exhibit lower water absorption values, as their larger pore volume drains the water after immersion. Currently, only a limited number of studies have examined the short-term water absorption behavior of loose-fill insulation materials; therefore, results from studies on mat-shaped or board-shaped bio-based insulation materials were also considered for comparison.

Comparable results were reported by Marques et al. [69], who measured a W_p_-value of 4.6 kg/m^2^ for untreated rice straw bales with a density of 91.7 kg/m^3^. Similarly, Zach et al. [78] investigated hemp fiber insulation mats bonded with bi-component fibers and various hydrophobic coatings, finding a short-term water absorption of 2.17 kg/m^2^ at a density of 38 kg/m^3^ for the untreated sample. In another study, Kremensas et al. [79] reported a W_p_-value of 4.42 kg/m^2^ for a hemp fiber composite with a density of 40 kg/m^3^. When compared to these materials, the treated leaf-based insulation in the present study demonstrates comparable or even superior short term water absorption performance, despite its substantially higher bulk density. This finding indicates a favorable water absorption behavior for the leaf material and suggests that the applied flame-retardant treatments may enhance hydrophobic properties without compromising structural integrity.

### 4.7. Hygroscopic Sorption Properties

Hygroscopic sorption properties describe a material’s ability to absorb and release water vapor from the surrounding environment. This characteristic plays a crucial role in regulating indoor humidity levels and thus contributes to the hygrothermal comfort and durability of building envelopes. The sorption isotherm of a material quantifies its moisture storage capacity, expressed as the moisture content at specific relative humidity (RH) levels and constant temperature. The moisture content of a material depends primarily on its hydrophilic or hydrophobic nature and its pore structure.

Thermal insulation materials based on natural fibers generally exhibit high hygroscopic capacity, which is mainly attributed to the high cellulose content of plant fibers. Cellulose is inherently hydrophilic due to the presence of hydroxyl groups capable of forming hydrogen bonds with water molecules [57,75,80,81]. In contrast, insulation materials produced from mineral- or fossil-based raw materials, such as glass wool, mineral wool, or expanded polystyrene, show very low moisture sorption ability [74,82,83].

In the present study, the untreated leaf material exhibited equilibrium moisture contents ranging from 5.8% at 30% relative humidity to 15.1% at 80% relative humidity. These values are consistent with the results reported for hemp and cellulose fiber insulation, which demonstrated similar moisture contents over the same humidity range [84]. This indicates that the leaf-based insulation material possesses moisture buffering characteristics comparable to other bio-based insulation materials, suggesting its potential to contribute to indoor humidity regulation in building applications.

## 5. Conclusions

This study demonstrated that urban leaf litter, when properly processed, represents a viable alternative raw material for loose-fill thermal insulation. The investigated material exhibited thermal conductivities between 0.041 and 0.046 W/m·K, comparable to other bio-based insulation products such as cellulose and wood fiber. A particle size fraction of 2–16 mm provided the best balance between thermal performance, dust generation, and processing behavior. Settlement tests confirmed that installation using pneumatic blowing allows compliance with the maximum settlement requirement of 1%. All investigated variants, including untreated material, achieved fire resistance class E, and selected flame-retardant treatments—particularly those containing calcium hydroxide and whey/sodium carbonate—further reduced flame spread. The material showed low water absorption and hygroscopic sorption properties comparable to other natural-fiber insulations, indicating good hygrothermal performance. The measured vapor diffusion coefficient of the developed materials ranges from 3.3 to 7.5, providing a favorable vapor transmission and reducing the risks of mold development. Resistance to mold growth was satisfactory under standardized conditions, though natural microbial variability warrants further investigation. Overall, leaf-based insulation offers an ecologically and economically attractive use for seasonal urban biomass, contributing to resource efficiency and circular economy objectives in the construction sector. Future work should address long-term durability, large-scale processing feasibility, and end-of-life treatment to support practical implementation.

## Figures and Tables

**Table 1:** Variants of leaf material with the particle size of 2–16 mm treated with different flame retardants in various concentrations.

Variant	Flame Retardant	Concentration (%)
R	-	-
A5	Ammonium phosphate	5
A10	Ammonium phosphate	10
A15	Ammonium phosphate	15
B5	Boric salt	5
B10	Boric salt	10
B15	Boric salt	15
K5	Calcium hydroxide	5
K10	Calcium hydroxide	10
K15	Calcium hydroxide	15
MS2/1	Whey/sodium potassium	2/1
MS4/2	Whey/sodium potassium	4/2
MS6/3	Whey/sodium potassium	6/3

**Table 2:** Mean values and standard deviation of thermal conductivity of different fractions.

Fraction	Thermal Conductivity Mean Value (W/m*K)	Thermal Conductivity Standard Deviation (W/m*K)
1–16	0.0414	0.00061
2–16	0.0423	-
0–16	0.0430	0.00076
2–4	0.0432	0.00123
2–6	0.0437	0.00078
8–10	0.0443	0.00050
4–6	0.0444	0.00062
1–2	0.0444	0.00050
6–8	0.0449	0.00137
10–12	0.0453	0.00100
0.5–1	0.0463	0.00050
8–16	0.0475	0.00139
0–2	0.0507	0.00116
0–0.5	0.0584	0.00031
16–31.5	0.0600	0.00086
uncrushed	0.0610	0.00200

**Table 3:** Mean values and standard deviation of thermal conductivity of different fractions, shown in Figure 3.

Fraction	Thermal Conductivity Mean Value (W/m*K)	Thermal Conductivity Standard Deviation (W/m*K)
2–4 E	0.0423	0.00060
2–4	0.0432	0.00123
4–6 E	0.0435	0.00033
4−6	0.0444	0.00062
6–8 E	0.0428	0.00036
6−8	0.0449	0.00137
1–16 E	0.0438	0.00029
1–16	0.0414	0.00061
2–16 E	0.0445	0.00060
2−16	0.0423	-
4−16	0.0439	0.00037

**Table 4:** Assessment of the mold growth on different variants (mean values, *n* = 3 for visual rating, *n* = 2 for number of fungal units) according to DIN EN 17886 and according to quantitative assessment after exposure at 28 °C ± 2 °C and 80 ± 5% RH.

Variant	DIN EN 17886 Assessment of Mold Growth According to DIN EN ISO 846:1997 Table 4	Number of Cultivable Fungal Units (log10 CFU/cm^3^)	
		Initial Load (T0)	Final Fungal Units (T4)
R	0	4.2	4.0
K5	1a	4.2	4.2
K10	1a	4.0	4.1
K15	1a	4.2	4.1
MS2/1	0	4.0	4.1
MS4/2	0/1a	4.2	4.1
MS6/3	0/1a	4.1	4.1
A5	0	4.2	4.0
A10	0	4.2	4.1
A15	0	4.3	4.2
B5	0	4.1	4.1
B10	0	4.1	4.1
B15	0	4.2	4.1

**Table 5:** Assessment of the mold growth on different variants (mean values, n = 5) according to EAD 040138-01-1201 after exposure at 23 °C ± 2 °C. R = untreated leaf material, RW = untreated leaf material washed two times, B5 = leaf material treated with 5% boric salt, SG = untreated seagrass, S = wheat straw (ISOSTROH loose fill insulation), Z = cellulose, HWF = wood fiber.

Variant	Assessment According to Table 4 DIN EN 846:1997	Bulk Density (kg/m^3^)
R	2	111
RW	2	110
B5	2	110
SG	2	45
S	2	100
Z	0	40
HWF	0	45

**Table 6:** Measured values of water vapor diffusion resistance for untreated (R.1–R.5) and with ammonium phosphate (A15.1–A15.5), a mixture of whey and sodium potassium (MS6/3.1–MS6/3.5), boric salt (B15.1–B15.5) and calcium hydroxide (K15.1–K15.5) treated material.

Sample	Water Vapor Transmittance Coefficient g (mg/m^2^h)	Permeability to Water Vapor W [mg/(m^2^*h*Pa)]	Water Vapor Diffusion Resistance Z ((m^2^*h*Pa)/mg)	Vapor Diffusion Resistance Factor µ (−)	Vapor Diffusion Resistance Factor MV µ (−)	Standard Deviation (−)
R.1	2409	1.80	0.6	4.2	4.7	0.591
R.2	1922	1.42	0.7	5.7		
R.3	1977	1.47	0.7	4.9		
R.4	2528	1.90	0.5	4.0		
R.5	2157	1.61	0.6	4.7		
A15.1	1278	0.91	1.1	8.1	7.5	0.942
A15.2	1292	0.92	1.1	8.1		
A15.3	1292	0.92	1.1	7.8		
A15.4	1781	1.27	0.8	5.6		
A15.5	1272	0.91	1.1	7.8		
MS6/3.1	2243	1.60	0.6	4.7	5.2	0.731
MS6/3.2	2107	1.50	0.7	5.2		
MS6/3.3	1736	1.24	0.8	6.4		
MS6/3.4	2432	1.74	0.6	4.3		
MS6/3.5	1925	1.37	0.7	5.4		
B15.1	1969	1.41	0.7	4.9	4.4	0.787
B15.2	2109	1.51	0.7	4.5		
B15.3	3337	2.38	0.4	2.9		
B15.4	1966	1.40	0.7	4.9		
B15.5	1848	1.32	0.8	5.0		
K15.1	3578	2.56	0.4	2.7	3.3	0.554
K15.2	2918	2.08	0.5	3.2		
K15.3	3381	2.41	0.4	2.8		
K15.4	2734	1.95	0.5	3.6		
K15.5	2184	1.56	0.6	4.2		

**Table 7:** Measured values of short-term water absorption by partial immersion (mean values, n = 4).

Variant	Bulk Density (kg/m^3^)	Short Term Water Absorption Wp (kg/m^2^)	Standard Deviation (kg/m^2^)
R	103	5.3	0.95
K5	96	3.3	1.27
K10	95	2.2	0.39
K15	101	4.3	1.02
MS2/1	106	4.0	0.67
MS4/2	103	4.4	0.87
MS6/3	103	4.4	0.85
B5	96	3.2	0.34
B10	101	2.9	0.29
B15	96	2.9	0.57
A5	95	3.8	0.14
A10	99	4.3	0.74
A15	102	4.3	0.57

**Table 8:** Water content (%M) at different relative humidities for treated and untreated leaf material (2–16 mm).

Variant	Water Content (%M) at a Relative Humidity of 30%	Water Content (%M) at a Relative Humidity of 40%	Water Content (%M) at a Relative Humidity of 50%	Water Content (%M) at a Relative Humidity of 60%	Water Content (%M) at a Relative Humidity of 70%	Water Content (%M) at a Relative Humidity of 80%
R	5.8	6.5	7.7	9.4	11.8	15.1
A5	5.5	6.3	7.5	9.1	11.4	14.6
A10	5.3	6.2	7.5	9.2	11.8	15.7
A15	4.8	5.6	7.0	8.7	11.3	18.8
B5	5.4	6.1	7.2	8.8	11.1	14.2
B10	5.4	6.2	7.5	9.2	11.8	15.3
B15	5.9	6.7	7.8	9.4	12.0	15.5
K5	5.8	6.5	7.4	9.0	11.7	15.1
K10	5.8	6.3	7.3	8.7	11.6	15.3
K15	5.5	6.1	7.1	8.6	11.5	15.5
MS2/1	6.3	7.0	8.2	9.9	12.5	16.1
MS4/2	5.2	6.0	7.3	9.0	11.6	15.7
MS6/3	5.5	6.1	7.2	8.9	11.7	15.7

## Data Availability

The original contributions presented in this study are included in the article. Further inquiries can be directed to the corresponding author.
